# Solar Energy and New Energy Technologies for Mediterranean Countries

**DOI:** 10.1002/gch2.201900016

**Published:** 2019-06-19

**Authors:** Rosaria Ciriminna, Lorenzo Albanese, Mario Pecoraino, Francesco Meneguzzo, Mario Pagliaro

**Affiliations:** ^1^ Istituto per lo Studio dei Materiali Nanostrutturati CNR via U. La Malfa 153 90146 Palermo Italy; ^2^ Istituto di Biometeorologia CNR via Madonna del Piano 10 50019 Sesto Fiorentino Italy; ^3^ via C. Giacquinto 1490135 Palermo Italy

**Keywords:** energy transition, Mediterranean, solar energy, utility‐scale photovoltaics

## Abstract

The replacement of fossil fuels to produce electricity and heat to propel vehicles with solar energy, along with other new energy technologies, will bring significant economic, environmental, and social benefits to all Mediterranean countries. The transition to new energy, however, calls for a proactive and creative role of policy makers.

## Introduction

Today's low‐cost, high efficiency, and huge and increasing production levels of photovoltaic (PV) solar modules (over 120 GW forecasted in 2019)^1^ render realistic to assume that all viable urban rooftops will be used to generate electricity. Seo and co‐workers have recently estimated that this would meet more than 60% of daylight electricity needs of cities in industrialized countries and over 30% of their all‐hours demand.^2^

Similarly, the low cost and pronounced versatility of today's solar thermal collectors (472 GW global capacity by early 2018)^3^ enables to use rooftops and facades to generate low temperature heat there where it is needed and consumed.^4^

Referring to Rome as the city with the world's largest historic heritage, and the consequent need for systematic architectural integration of the solar energy technology to combine historic preservation with generation of renewable energy, we have lately shown how today's versatile photovoltaic and solar thermal technology solutions enable to achieve the uptake of decentralized solar energy systems in practically any building and built environment.^5^

From Sicily^6^ through Spain,^7^ numerous studies have addressed the topic of building‐integrated solar technology in the Mediterranean area.^8^

Approaching the end of 21st century second decade, architects and engineers working in Mediterranean countries will have no difficulty to access updated books^9^ and advanced studies^4^, ^10^, ^11^ on solar architecture to learn how to effectively integrate solar energy technology into the architectural envelope.

Alongside distributed generation via rooftop solar systems, ground‐mounted large PV arrays are instrumental for the transition from fossil fuel to renewable electricity. From Morocco through Algeria, Egypt, and even Syria, where a 1.26 MW ground‐mounted solar power plant near Damascus started operating in late 2018, low levels of investment in large PV parks in southern and eastern Mediterranean countries are finally behind us.

By the end of 2018, 2.9 GW of solar PV were operating in the Middle East and North Africa area, with 12 GW of solar projects under construction or awarded.^12^

The countries in the southern and eastern Mediterranean region (Morocco, Algeria, Tunisia, Lybia, Egypt, Israel, Palestinian Territories, Lebanon, Syria, and Turkey) have huge solar irradiation levels, but almost invariably they rely on fossil fuels to meet almost all of their energy demand. The latter demand, furthermore, is rapidly growing alongside population and living standards making these countries the ideal location for the large‐scale development of solar PV.

Calling for a proactive and creative role of policy makers, this study aims to show how and why the replacement of fossil fuels with solar energy to produce electricity, heat, hot water, and propel vehicles, will bring significant economic, social, and environmental benefits to all Mediterranean countries.

## The Energy, Population, and Wealth Trilemma

In order to feed the growth of the gross domestic product (GDP) identified by a recent mathematical model combining the global population, wealth, and oil consumption,^13^ the world's energy consumption by 2025 should increase by about 1700 million tons of oil equivalent (MTOE) per year.

This means that even to keep the oil fraction in the energy mix at the 2015 low level of around 33%, the exceptionally high amount of more than 11 additional million barrels of oil per day should be added to current production levels.

Algeria, a large producer and exporter of natural gas, nicely renders the urgency of the transition to solar energy to face the energy, population, and wealth trilemma identified above.^13^

The country has more than doubled its energy consumption from 28 MTOE in year 2000 to 58 MTOE in 2015, while the GDP at constant prices increased by more than 70%.^14^ The Algeria's economy in 2017 consumed about 78 TWh of electricity. The figure is forecasted to increase to 123–160 TWh a^−1^ by 2025.^15^

Currently 97% of the country's electricity demand is met by burning natural gas.^15^ However, dramatic growth in energy consumption combined with the fall in production led to a nearly 31% drop in natural gas exports between 2015 and 2005.^14^ These figures explain the urgency for Algeria to uptake utility‐scale PV generation, whose low cost and high reliability enable the production of electricity sold at a profit for $0.03/kWh and even less.^16^

From Morocco to Syria, the countries of the southern and eastern Mediterranean basin share exceptional insulation levels, which make them ideally suited for the deployment of utility‐scale PV. Indeed most governments of said countries in the course of the last 2 years launched international auctions for procurement of large amounts of PV electricity via power purchase agreement (PPA) contracts typically lasting 15–20 years. Regardless of the country, the tender selection process is based on the lowest bid from financially robust and technically advanced renewable energy companies which are first “shortlisted” and then selected to build, own, and operate the plant for the whole duration of the PPA.

For example, the Egypt's 200 MW tender for the Kom Ombo solar project in early August 2018 attracted bids as low as $27.52/MWh ($0.02752 kWh^−1^), which led the Egyptian Electricity Transmission Company to set a maximum price of $0.025 for a forthcoming tender for 600 MW of PV capacity in the west of Nile area.^17^

Algeria hosts its first significant PV plant since early 2018, when the 233 MW solar park comprised of 16 different PV plants in different areas of the Adrar province was connected to the grid. The parks, whose construction started in January 2016 and was completed in January 2018, were built by a consortium of Chinese companies which included the module supplier.

Algeria currently hosts only 500 MW of PV power. Out of the 22 GW of new renewable energy power expected by 2030 according to the country's *Plan National de Développement des Energies Renouvelables*, 13575 MW should originate from solar PV and 5010 MW from wind power.^18^

It is instructive to learn that the tender issued on November 2018 by the Algerian Electricity and Gas Regulation Commission for the construction of several PV power plants with a combined capacity of 150 MW in southwestern Algeria required the use of PV modules assembled in Algeria as well as locally manufactured mounting structures and cables.^19^

In brief, besides decoupling electricity production from natural gas consumption, Algeria is using part of the revenues originating from the exports of natural gas to incentivize the foundation of the new solar energy industry in the country.

Indeed, according to the entrepreneur president of Algerian Solar Energy Association talking to the specialized press in mid 2018:

“There is now a solid solar manufacturing industry base under development in Algeria. We estimate that around 550 MW of solar module assembly capacity will be operational in the country by the end of this year. Furthermore, there are already several manufacturers of various parts of the supply chain, such as junction boxes, mounting structures and cables, PV glass, aluminum frames, which are able to provide a wide range of high quality products.”^20^

Similar tenders for utility‐scale PV projects were lately undertaken by the Tunisian, Egyptian, and Moroccan governments with several solar projects under development and construction.

The number and size of these projects will only expand due to overwhelming abundance, low, and declining cost of PV modules and inverters as well as to straightforward installation of huge solar parks.

**Figure 1:**
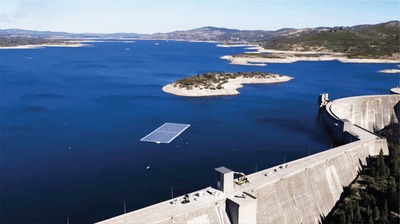
The 218 kW floating solar plant on Portugal's Alto Rabagao hydroelectric dam. Connected to the grid in late 2016, the floating system supports 840 PV modules, with bottom anchoring designed to meet a maximum depth of 90 m and a level variation of 30 m. Photo reproduced with permission from Ciel & Terre.

Furthermore, as it will shortly happen in Malta (a 5 MW plant to be installed over the large landfill closed in 2013), utility‐scale PV plants will be ideally installed on the surface of closed landfills^21^ as well as floating on dam, reservoirs, pond, and artificial lake water surface (a symbiotic technology pioneered in France and in Italy, now widespread across the world, **Figure** **1**).^22^

What is emphasized herein is that North Africa Mediterranean countries are finally expanding their energy generation mix with renewable energy, aiming at the same time to expand their manufacturing industry by attracting new investments from foreign companies specializing in new energy technologies.

**Figure 2:**
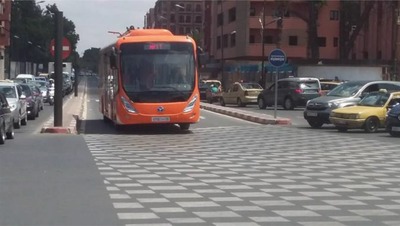
The roads of Marrakech, Morocco, have hosted 15 electric buses since 2017. Each bus runs with overhead cables outside the city center but each bus runs only on battery energy within the city. Photo reproduced with permission from https://www.skyscrapercity.com/showthread.php?t=1468062&page=20.

Morocco, for example, in 2017 became the first North African country to host a fleet of 15 electric buses now running through the streets of Marrakesh where they transport over 45 000 people each day, reducing air pollution and improving the quality of the transportation service (**Figure** **2**).

In the same year the country's government signed an agreement with one of the world's largest manufacturers of battery electric vehicles to build a new plant within the new industrial city of Tangier where battery electric vehicles, including e‐buses, will be manufactured.^23^

## Distributed Generation

Alongside utility‐scale PV plants, in Mediterranean countries the adoption of distributed generation via buildings functionalized with solar PV and solar thermal collectors will shortly accelerate driven by the low cost of today's solar technology and by its newly developed aesthetic value^4^, ^5^ as well as from new energy technologies, such as the lithium‐ion battery for electricity storage and light‐emitting diodes for lighting.

There is no reason for Mediterranean cities and regions to continue to pay ever‐costlier electricity bills to light streets and outdoor environments when today's solar street light emitting diode (LED) lights equipped with smart battery and lighting management systems provide reliable, quality lighting at a fraction of the cost of conventional technology, both in developing and developed countries.^24^

**Figure 3:**
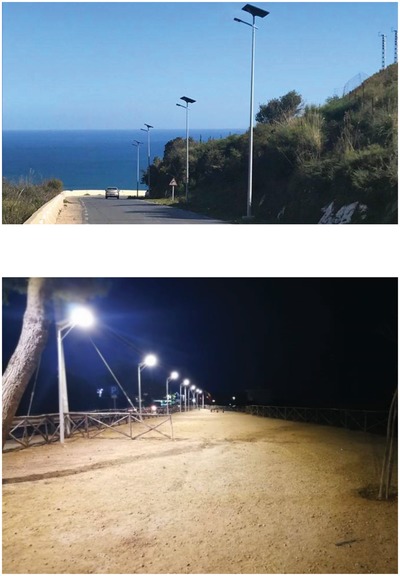
Since mid‐2018, solar street lights have lit the 8 km road connecting Annaba, Algeria, to its beaches (top photo reproduced with permission from Fonroche Eclairage) and the belvedere sulla Scala dei Turchi in Realmonte, Sicily (bottom photo reproduced with permission from Noon Technologies).

For example, the 8 km coastline road connecting the city of Annaba in northeast Algeria to its fine beaches is now lit with more than 200 off‐grid solar streetlights (**Figure** **3**) generating an average of 13 lux on the ground thanks to the white light (4000 K color temperature) emitted by an impermeable 40 W LED fixture emitting 160 lm W^−1^.^25^

The electric current is supplied by a battery charged during the day by the current originating from the PV module in polycrystalline silicon placed on the top of a 5 m pole of steel specially treated to withstand corrosion in the salty seaside environment. The system is supplied with a 5 year warranty, with 10 year lifespan without maintenance.

The city does not receive any electricity bill, whereas the road safety dramatically improved as the twisty road previously had remained unlit (mostly due to the high cost to lit coastal roads with their constant bends and undulating elevation).

Similar solar streetlights already lit many areas in Egypt, including all main streets in Sharm El‐Sheikh, and are being installed at fast pace in southern and eastern Mediterranean countries.

The key point is that today these technologically advanced off‐grid lights can conveniently replace most conventional lights altogether, getting rid of electricity lighting bills. As of early 2019, for example, India was installing 3 million solar street lights across the country paying each light approximately $356.^26^

Hence, in the Mediterranean island of Sicily where most municipalities face difficulties to pay the public lighting electricity bill, uptake of the economically viable and reliable solar street lighting will be rapid, as in the recent case of the belvedere in the southern Sicily's town of Realmonte (Figure 3) now lit with elegant “all‐in‐one” solar lights equipped with LED fixture emitting each 7900 lumen of white light with the appropriate 3500 K color temperature.^27^

**Figure 4:**
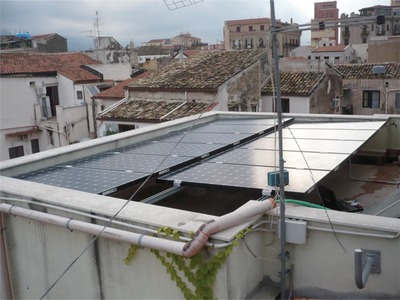
PV array on the rooftop of a building in the old city's center of Palermo, Sicily. Photo reproduced with permission from Medielettra.

By the same token, today's efficient and aesthetically pleasing solar thermal or PV modules are increasingly applied to existing rooftops and façades of Mediterranean region buildings maintaining the character of the local built environment by meeting a few important requirements of place‐responsive design aimed at improving the visual impact (the effect on views and visual amenity as experienced by people).^28^ For instance, the use of nonreflective black modules with a black frame makes the overall PV module array more discrete by diminishing the contrast with the roof (**Figure** **4**).

Ten years ago, PV modules in polycrystalline silicon (p‐Si) were significantly cheaper than modules employing monocrystalline Si solar cells. Still, responsive design encouraging an approach “sympathetic to the visual appearance of the building and the local area from which it can be seen”^28^ resulted in excellent realizations in Mediterranean urban areas even when using the blue p‐Si modules.

**Figure 5:**
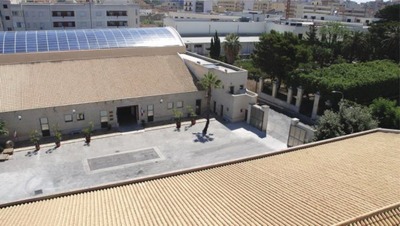
The rooftop of the Pellegrino winery in Marsala, Sicily. Photo reproduced with permission from Cantine Pellegrino.

For example, the coplanar siting of the modules on the rooftop retains the original shape of the roof (**Figure** **5**) regardless of its shape (flat, sloped or curved roof), thanks to not visible racking system.

Today's modules best suited for residential and commercial rooftop applications typically employ high‐efficiency m‐Si cells using passivated emitter rear cell (PERC) technology with five (or even more) silver bus bars, or even eliminating bus bars and ribbon interconnections altogether by adopting the back contact technology.

**Figure 6:**
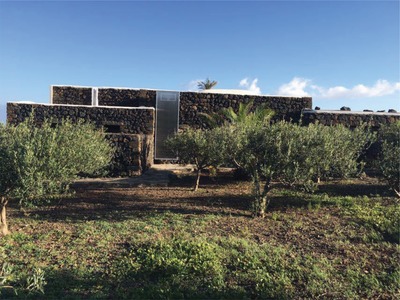
A typical home on Italy's island of Pantelleria, Sicily, with a solar air collector integrated on the south façade.

Similar progress has concerned solar thermal collectors. **Figure** **6**, for instance, shows a typical home in Sicily's island Pantelleria whose south façade built in local lavic stone is functionalized with a solar air collector.

Fully meeting the performance expectations of the poorly known yet highly efficient solar air heating technology,^29^ the home has lost in a few days following the installation of the light solar air collector (a polycarbonate glazing over black aluminum selective absorber) in Autumn 2017, the high indoor relative humidity levels that plague practically all buildings in the numerous islands of the Mediterranean sea. The average indoor temperature has increased by more than 3 °C and residents claiming to enjoy comfort thought “not possible” before.

Sicily alone is surrounded by 14 inhabited islands whose population consumes significant amounts of fossil fuel to produce electricity and heat when practically all energy demand could be met by a balanced mix of utility‐scale PV coupled to storage in Li‐ion batteries, and distributed generation via PV and solar thermal collectors integrated into buildings.^30^

## Conclusions and Recommendations

Calling for a proactive and creative role of policy makers in different Mediterranean countries, this study aims to show how the replacement of fossil fuels employed to produce electricity, heat and to propel vehicles with solar energy and new energy technologies may bring significant economic, environmental, and social benefits.

The outcomes of the first decade of the great energy transition to renewable energy and to new energy technologies, indeed, are clear:

- Utility‐scale photovoltaic power generation is now so cheap that one MWh from a large PV park in Egypt is profitably sold at $27.52;^17^
- PV and solar thermal technologies are ready to functionalize all sort of buildings at low cost and produce useful energy on‐site;^4^, ^5^
- Battery electric vehicles are ready to replace internal combustion vehicles burning oil;^31^

Under these circumstances, policy makers in Mediterranean countries need to focus policies and public investment to achieve three main objectives: i) to bring the benefits of the new energy technologies to citizens via accelerated uptake of distributed generation, ii) incentivize the deployment of utility‐scale PV parks, preferably including storage, and iii) to ease the opening of new energy technology factories in their countries.

Today's low cost solar energy and electricity storage technology, make the first objective achievable thanks to new legislation supporting distributed generation for example updating obsolete construction regulation making long and costly the permit process to functionalize buildings with solar modules.^5^ The growth potential is huge as less than 5% of the world's buildings, and even a lesser fraction of the large Mediterranean built environment, are functionalized with solar collectors.^32^

Achieving the second objective (large PV plants and large PV plants including storage) in industrialized countries such as Spain, Italy, and France is feasible by allowing renewable energy generation companies to take part not only to the day‐ahead electricity market (where they already significantly reduce the wholesale price)^33^ but also to the dispatching and energy services markets, so far opened to thermal production units only.

Sunnier Mediterranean African countries will preferably continue with tenders to purchase clean electricity produced via utility‐scale PV plants at prices that in the case of Egypt have already reached the aforementioned (and astonishing) low level of $27.52 MWh^−1^.^17^

Finally, the third and most challenging objective, to make their countries home to new industrial plants of the solar economy, requires to concentrate the financial resources on establishing new partnerships with the leading renewable energy and energy storage technology manufacturers, which are not based in Mediterranean countries.

There are many ways to incentivize foreign companies to invest, as shown by Morocco with the new electric vehicle (EV) plant due to start soon; by Algeria, requiring renewable energy companies to use components made in Algeria for the utility‐scale PV plants awarded the PPAs; or by Turkey where construction of a 500 MW manufacturing facility of PV products, including silicon ingots, wafers, cells, and modules, is approaching completion in Ankara in co‐operation with a South Korea‐based solar cell manufacturer under a partnership contract awarding $69.9 MWh^−1^ for an energy purchase term of 15 years to the consortium that will build the 1000 MW solar park in the Konya Province.^34^

In conclusion, following the example of Morocco and aware that renewed education in solar energy and new energy technologies is a key enabler of economic development,^35^ Mediterranean county policy makers will establish new solar energy research and educational institutes where to shape the professionals needed to guide and ease the energy transition.^36^

## Conflict of Interest

The authors declare no conflict of interest.
